# Numerical Investigation of the Axial Compressive Behavior of a Novel L-Shaped Concrete-Filled Steel Tube Column

**DOI:** 10.3390/ma18040897

**Published:** 2025-02-19

**Authors:** Fujian Yang, Yi Bao, Muzi Du, Xiaoshuang Li

**Affiliations:** 1 School of Urban Construction, Changzhou University, Changzhou 213164, Chinayibao10@126.com (Y.B.); 2 Key Laboratory of Rock Mechanics and Geohazards of Zhejiang Province, Shaoxing 312000, China; 3 CCCC Water Transportation Consultants Co., Ltd., Beijing 100007, China; 4 College of Civil Engineering, Qilu Institute of Technology, Jinan 250200, China

**Keywords:** L-shaped column, CFST columns, axial ratio, stress distribution, confinement effect

## Abstract

A novel L-shaped concrete-filled steel tube (CFST) column is proposed in this study. A finite element model of the column is developed using ABAQUS software to analyze its load transfer mechanism and axial compressive behavior. The effects of factors such as the steel strength, steel tube thickness, support plate configuration, and perforation of the support plates on the compressive performance of the column are investigated. The simulation results reveal that the column exhibits robust axial compressive performance. Increasing the steel strength and incorporating support plates (SP) effectively enhance the column’s compressive bearing capacity and positively influence the bearing capacity coefficient (δ). However, increasing the steel tube thickness results in a reduction in δ, indicating that the rate of increase in the bearing capacity diminishes with increasing thickness. The failure mode is primarily characterized by local buckling in the midsection of the steel tube’s concave corner. Measures such as increasing the steel strength and tube thickness and the use of support plates help to mitigate buckling at the concave corner, improve concrete confinement, and enhance the overall compressive performance of the column.

## 1. Introduction

In recent years, with increasing governmental advocacy and support for green building initiatives, low-carbon, environmentally friendly, and sustainable development principles have become pivotal in modern design and construction practices. Against this backdrop, concrete-filled steel tube (CFST) structures have emerged as an innovative solution that seamlessly integrates the strengths of both concrete and steel, offering superior load-bearing capacities and exceptional ductility. Compared to structures composed solely of steel or concrete, CFST systems exhibit significantly enhanced load-bearing performance, often exceeding the combined strength of their constituent materials [1,2,3]. This structural form [4,5,6] excels in resisting complex load scenarios and demonstrates remarkable mechanical performance and durability under extreme environmental conditions such as earthquakes [7,8] and tsunamis. These attributes not only ensure the stability and integrity of building structures but also significantly extend their service life, underscoring the potential of CFST structures in sustainable construction.

**Figure 1:**
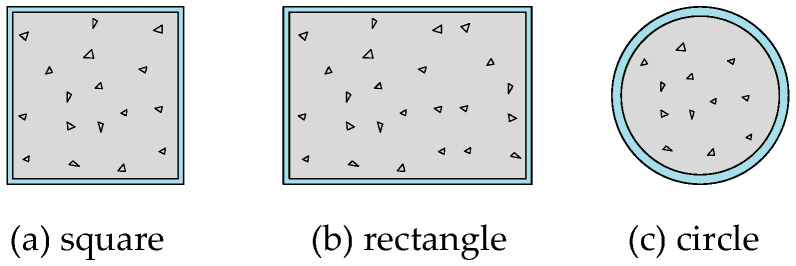
Cross-sectional forms of traditional CFST columns.

Traditional CFST columns commonly feature rectangular, square, or circular cross-sections, as illustrated in Figure 1 [9]. These conventional cross-sectional forms are extensively utilized in construction due to their simplicity and efficiency. However, they also present certain limitations in practical applications, such as large volumes, construction constraints, and challenges associated with protruding corners. These issues can hinder the realization of flexible spatial layouts, particularly in spaces with unique or irregular configurations. To address these challenges, uniquely shaped CFST columns have been developed. With their adaptable design characteristics, these columns offer versatile solutions that cater to diverse building structures and spatial layouts, especially in architecturally unconventional or irregular spaces. Additionally, their distinctive forms contribute to a sense of modernity and artistic appeal, enhancing the overall aesthetic value of the building. Commonly used uniquely shaped CFST columns in contemporary construction include T-shaped, cross-shaped, and other specialized configurations, which have proven effective in balancing functional and aesthetic considerations.

Despite the numerous advantages of irregular CFST columns, there are still some challenges, such as thin column limbs, excessive aspect ratios, stress concentration at the corners, and the weak constraint effects of the steel tube on the core concrete. To address these issues, several scholars have proposed improvements to optimize the design of irregular columns. To enhance the performance of traditional T-shaped CFST columns, Tu et al. [10] introduced a multi-unit T-shaped CFST (MT-CFST) column structure. Their study involved experimental tests on 13 short specimens with various cross-sectional shapes and material properties, as well as 12 slender specimens with different length-to-thickness ratios under an axial load. The study comprehensively explored the mechanical behavior of the multi-unit T-shaped CFST columns, focusing on the failure modes of short specimens, the relationship between the axial load and strain, and the axial load-bending curves of slender specimens. Liu et al. [11] conducted axial compression tests on L-shaped and T-shaped CFST columns to investigate the mechanical performance of uniquely shaped CFST short columns. During the experiments, detailed observations were conducted on the specimens’ behavior and failure modes. The results showed that reinforcement ribs effectively delayed the local buckling of the steel tube, significantly improving the tube’s buckling load-bearing capacity and enhancing its constraint effect on the concrete. Liu et al. [12] designed and fabricated 11 L-shaped multi-unit CFST short columns for axial compression tests, exploring the effects of the column side plate width-to-thickness ratio and concrete compressive strength on the failure mode, load-bearing capacity, and ductility of the specimens. The test results indicated that multi-core steel tubes significantly improved the constraint effect on the concrete. As the width-to-thickness ratio of the column side plates increased, the ductility gradually decreased. Moreover, increasing the compressive strength of the concrete enhanced the load-bearing capacity of the specimens but reduced their ductility. Hasan et al. [13] conducted axial compression tests on three half-scale cross-shaped CFST columns, evaluating their failure modes, stress, displacement, and ductility. They also proposed a method to determine the maximum load-bearing capacity of cross-shaped CFST columns. This method provides guidance for the design of irregular cross-shaped CFST columns based on different seismic fortification requirements.

L-shaped CFST columns have gained attention due to their unique structural geometry, which enhances their adaptability to diverse spatial layouts and makes them particularly suitable for complex architectural designs. Although preliminary investigations into the mechanical behavior of L-shaped CFST columns have been conducted in recent years, several critical findings have emerged. Han et al. [14] performed quasi-static tests to analyze how the geometric dimensions and steel tube configurations affect the seismic performance, revealing the critical role of the web thickness-to-flange length ratios in the energy dissipation capacity. Through parametric studies, Liu et al. [11] demonstrated that controlling the steel plate width-to-thickness ratio and core concrete compressive strength could significantly improve the ultimate bearing capacity and ductility coefficients. Du et al. [15] further validated the enhanced ductility of multi-cavity designs by establishing a trilinear skeleton curve model considering the slenderness ratio and axial compression ratio coupling effects, based on cyclic loading tests of nine L-shaped CFST specimens. However, existing research predominantly focuses on macroscopic parameter optimization and global response analysis. The refined design of internal stiffener configurations—including the quantity, layout patterns, and perforation parameters—and their influence on the localized stress distribution and buckling suppression mechanisms remain to be fully revealed. This knowledge gap limits the engineering application of such components in complex loading scenarios.

Therefore, this study proposes a novel L-shaped CFST column design featuring internal support plates to address challenges such as stress concentration and local buckling. Using the ABAQUS finite element software, nine L-shaped CFST column models were developed and analyzed to obtain load–displacement curves. The study systematically investigated the effects of the steel tube thickness, the number and configuration of internal support plates, the perforation settings, and the presence of holes on the compressive performance of the columns. Furthermore, the stress distribution of the support plates at the maximum displacement of the column ends was examined to provide insights into their contribution to the structural behavior. This research aims to advance the understanding and optimization of L-shaped CFST columns, contributing to their broader application in engineering practice.

## 2. Structural Forms of L-Shaped CFST Columns

**Figure 2:**
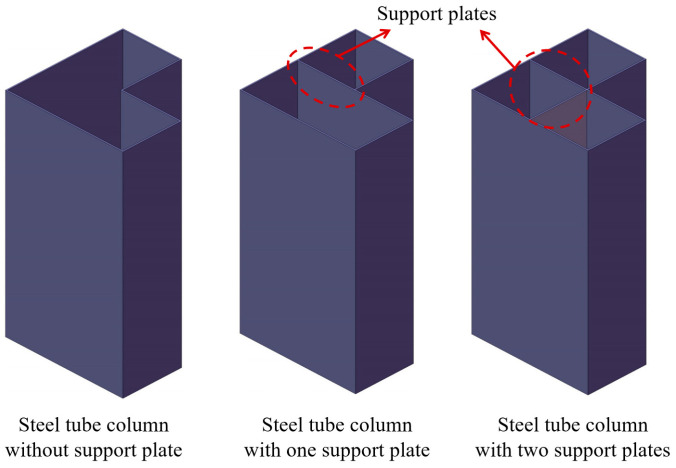
Schematic of steel tube column.

**Figure 3:**
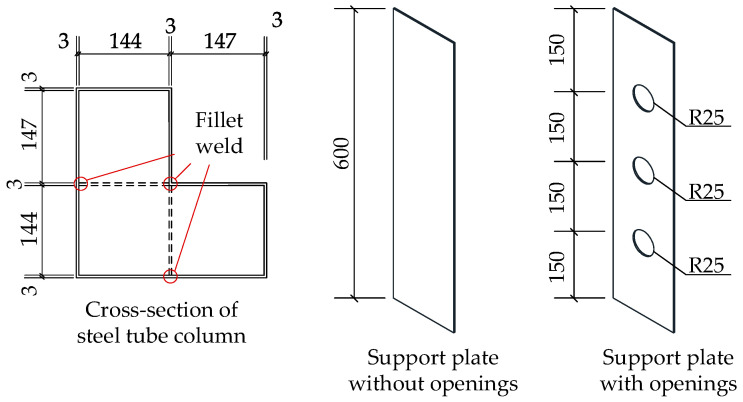
Dimensional design of L-shaped steel tube column.

Figure 2 illustrates the cross-sectional configuration of the proposed novel L-shaped CFST column. In this design, support plates are fillet-welded to the corners of the L-shaped steel tube, forming an integrated structure. The concrete used is C40-grade commercial concrete. For L-shaped CFST columns without support plates, concrete is directly poured into the steel tube cavity. In contrast, columns equipped with support plates have their steel tube cavities divided into multiple independent chambers, necessitating separate concrete pouring for each chamber. In columns featuring perforated support plates, the holes act as connecting passages between the chambers, enabling single-step concrete pouring into the entire cavity. This study focuses on short L-shaped CFST columns, with the CFST-LH1 model having a column height of 800 mm, while all other models have a height of 600 mm. The cross-sectional dimensions of the columns are detailed in Figure 3.

## 3. Numerical Modeling of L-Shaped CFST Short Columns

### 3.1. Finite Element Models

In this investigation, nine finite element models of innovative L-shaped CFST columns were developed using the ABAQUS software [16] to conduct a comprehensive numerical analysis. The study examined the effects of key factors, including the steel tube thickness (*t_st_*), the material strength, the presence and configuration of internal support plates (SP), and the setting of holes in the support plates. The geometry of the L-shaped steel tube sections was defined as a long side measuring 300 mm and a short side of 150 mm, as illustrated in Figure 3. The top and bottom end plates were designed as squares with a side length of 400 mm and a thickness of 20 mm. The specific characteristics and configurations of the specimens are summarized in Table 1.

### 3.2. Material Properties

#### 3.2.1. Concrete Material

**Figure 4:**
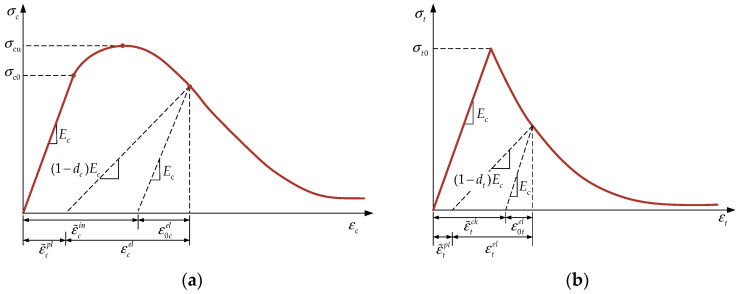
Constitutive models for the CDP: (**a**) compressive; (**b**) tensile.

In finite element analysis, the accuracy of the material constitutive models and the precision of the parameter inputs are critical factors in ensuring reliable simulation results. The constitutive models available in ABAQUS for the simulation of concrete include the Concrete Damage Plasticity (CDP) model, the Discrete Cracking model, and the Brittle Cracking model. In this study, the CDP constitutive model (Figure 4) is chosen due to its ability to effectively capture the behavior of confined concrete. The CDP model incorporates a concrete damage factor, accounting for the accumulation of damage as the material is loaded, which makes it particularly suitable for the simulation of the stress state of confined concrete. Given the relatively weak confinement of the core concrete by the steel tube in L-shaped CFST columns, the uniaxial compressive stress–strain relationship for concrete, as specified in GB50010-2010 [17], is adopted for this analysis.

(1) $$\sigma_{c}=(1-d_{c})E_{c}\varepsilon_{c}$$

(2) $$d_{c}=\left\{\begin{array}{ll} 1-\rho_{c}n/(n-1+x^{n}) & \left(x\leq 1\right)\\ 1-\rho_{c}/\left[\alpha_{c}{\left(x-1\right)}^{2}+x\right] & \left(x>1\right) \end{array}\right.$$

(3) $$x=\varepsilon_{c}/\varepsilon_{c,r}$$

(4) $$\rho_{c}=f_{cm}/\left(E_{c}\varepsilon_{c,r}\right)$$

(5) $$n=E_{c}\varepsilon_{c,r}/\left(E_{c}\varepsilon_{c,r}-f_{cm}\right)$$

where *E_c_* is the elastic modulus of concrete; *σ_c_* is the compressive stress of concrete; *ε_c_* is the compressive strain of concrete; *ε_c,r_* is the peak compressive strain of concrete; *f_cm_* is the average value of the axial compressive strength of concrete prisms from the test; *α_c_* is the parameter value for the descending segment of the uniaxial compressive stress–strain curve of concrete; and *d_c_* is the damage evolution parameter for uniaxial compressive concrete.

The tensile constitutive relationship of concrete is selected from the uniaxial tensile stress–strain curve of concrete in GB50010-2010 [17]:

(6) $$\sigma_{t}=(1-d_{t})E_{c}\varepsilon_{t}$$

(7) $$d_{t}=\left\{\begin{array}{ll} 1-\rho_{t}(1.2-0.2x^{5}) & \left(x\leq 1\right)\\ 1-\rho_{t}/\left[\alpha_{t}{\left(x-1\right)}^{1.7}+x\right] & \left(x>1\right) \end{array}\right.$$

(8) $$x=\varepsilon_{t}/\varepsilon_{t,r}$$

(9) $$\rho_{t}=f_{tm}/(E_{c}\varepsilon_{t,r})$$

where *σ_t_* is the tensile stress of concrete; *ε_t_* is the tensile strain of concrete; *ε_t_*,*_r_* is the peak tensile strain of concrete; *f_tm_* is the average value of the axial tensile strength of concrete from the prism test; *α_t_* is the parameter for the descending segment of the uniaxial tensile stress–strain curve of concrete; and dt is the damage evolution parameter for uniaxial tensile concrete.

**Figure 5:**
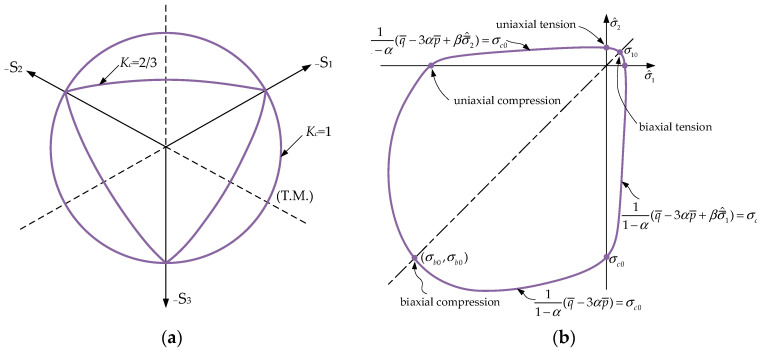
Typical yield surfaces for the CDP: (**a**) yield surfaces in the deviatoric plane; (**b**) yield surface in plane stress.

The CDP model adopts a yield criterion based on the function proposed by Lubliner et al. [18], with subsequent modifications by Lee and Fenves [19] to capture the distinct evolution of the strength under tension and compression. Typical yield surfaces are illustrated in Figure 5, which shows the yield behavior both in the deviatoric plane and under plane stress conditions. The detailed theoretical formulas and algorithms can be found in references [18,19].

#### 3.2.2. Steel Material

**Figure 6:**
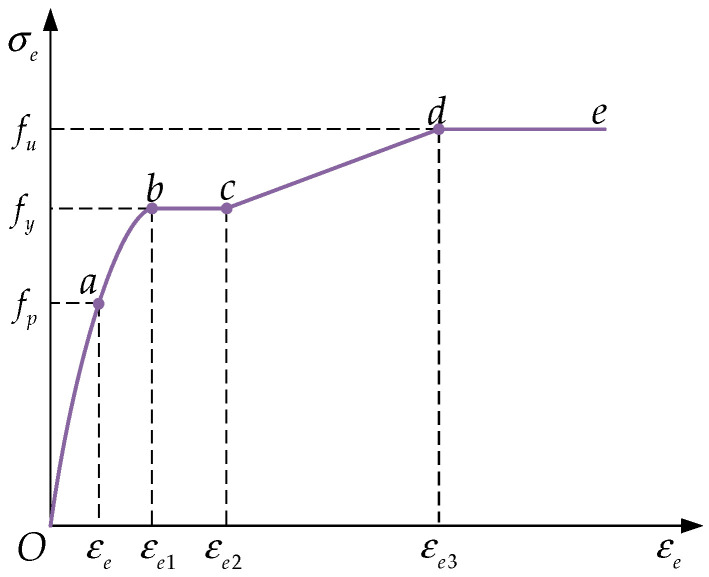
Stress–strain curve of steel.

The constitutive model for steel is based on the stress–strain curve for low-carbon steel proposed by Han [20,21,22], as shown in Figure 6. The curve is divided into five stages: the elastic stage (*Oa*), elastoplastic stage (*ab*), plastic stage (*bc*), strain hardening stage (*cd*), and secondary plastic flow stage (*de*). The Bauschinger effect is considered by setting isotropic hardening in the ABAQUS software. To simplify the calculations, the strain hardening stage is assumed to follow a linear relationship, and a horizontal plastic stage is set when the ultimate strength is reached. The stress–strain relationship in Figure 6 represents the simplified version of this curve. The mathematical expression of this model is given by Equation (10).

(10) $$\sigma_{s}=\left\{\begin{array}{l} E_{s}\varepsilon_{s}\\ -A\varepsilon_{s}^{2}+B\varepsilon_{s}+C\\ f_{y}\\ f_{y}\left[1+0.6\frac{\varepsilon_{s}-\varepsilon_{e2}}{\varepsilon_{e3}-\varepsilon_{e2}}\right]\\ 1.6f_{y} \end{array}\right.$$

where *f_y_* represents the yield strength of the steel; *E_s_* is the elastic modulus of the steel; and *ε_e_* is the strain corresponding to the proportional limit, *ε_e_*_1_ is the strain corresponding to the yield strength, *ε_e_*_2_ is the strain at the end of the yield stage, and *ε_e_*_3_ is the strain corresponding to the ultimate strength. The parameters *A*, *B*, and *C* in Equation (10) are defined as follows:

(11) $$A=0.2f_{y}/{\left(\varepsilon_{e1}-\varepsilon_{e}\right)}^{2}$$

(12) $$B=2A\varepsilon_{e1}$$

(13) $$C=0.8f_{y}+A\varepsilon_{e}^{2}-B\varepsilon_{e}$$

### 3.3. Mesh Generation and Definition of Contact Relations

**Figure 7:**
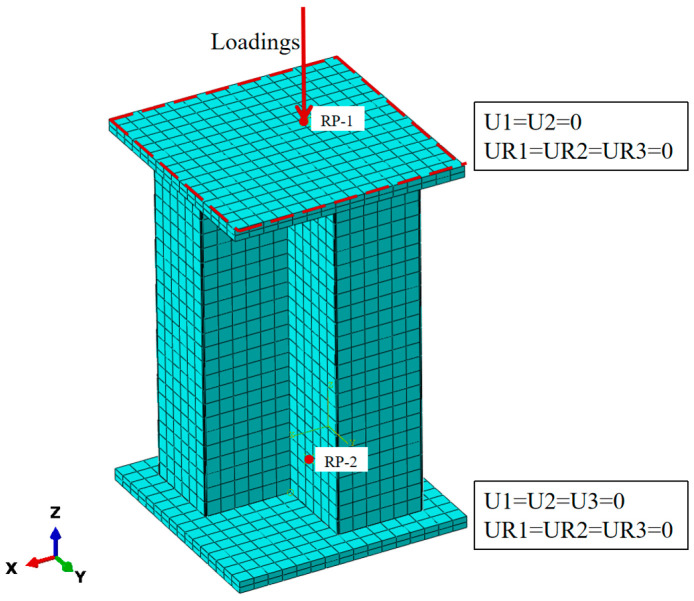
Finite element model of irregular L-shaped CFST column.

In this study, the finite element model of the L-shaped CFST column is constructed using C3D8R elements to simulate the steel tube, support plates, and concrete components. The meshing process is executed with the structured adaptive meshing method in ABAQUS, with each component independently meshed. To enhance the accuracy and computational efficiency of the finite element analysis, consistent seed densities are applied at the contact interfaces between components. The mesh distribution for the overall structure and individual components is presented in Figure 7.

The contact interactions between the loading plate and the upper and lower ends of the steel tube are defined as “tie” constraints. The normal interaction between the steel tube and the concrete is modeled using the “hard” contact model. For comparison with experimental results, the tangential interaction is defined using the penalty contact model with a friction coefficient of 0.6 [23,24,25]. However, during parameter analysis, tangential contact is defined as “frictionless”. This approach is adopted because excluding tangential friction in nonlinear analysis provides more accurate results.

### 3.4. Verification of Finite Element Modeling Method

**Figure 8:**
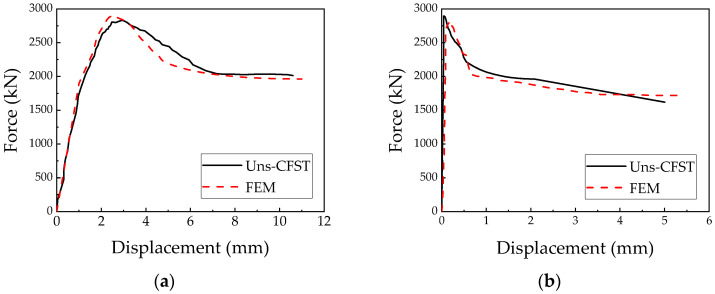
Load–displacement relationship comparison for cross-shaped CFST column: (**a**) load–vertical displacement relationship curve of CFST column; (**b**) load–horizontal displacement relationship curve at midsection of CFST column.

The finite element modeling approach proposed in this study was validated using a specimen from reference [13]. The concrete, cover plates, and steel tube columns were all modeled using eight-node reduced-integration 3D solid elements (C3D8R). To maintain mesh consistency among the different materials and reduce potential stress concentration at the interfaces, the element size for both the concrete and cover plates was set to 20 mm, with the steel tube also meshed at 20 mm, ensuring the overall accuracy and computational efficiency of the analysis. As illustrated in Figure 8, the finite element analysis curve aligns closely with the corresponding experimental curve. Under axial loading at the top of the column, the simulation results reveal that the vertical displacement of the steel tube column and the horizontal displacement at the column’s mid-span exhibit comparable development trends. At the maximum displacement, the regions of the steel tube with significant equivalent plastic strain (PEEQ) correspond well to the bulging locations observed in the experiment. The difference in peak load between the finite element simulation and the experimental results for the cruciform CFST column is approximately 3.24%, demonstrating the accuracy of the finite element modeling approach for cruciform CFST columns under axial compression and validating the effectiveness of the proposed method.

## 4. Performance Analysis

### 4.1. Performance Analysis Under Axial Compression Loading

**Figure 9:**
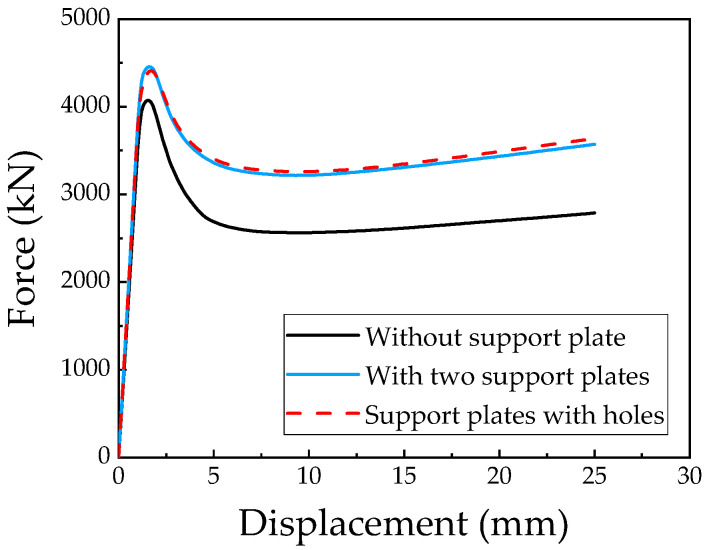
Load–displacement relationships for L-shaped CFST columns with different structural forms.

Figure 9 presents the load–displacement curves for the CFST-M-2, CFST-S-2, and CFST-S-3 specimens. The numerical results indicate that the peak loads for the CFST-S-2 specimen, which includes two support plates, and the CFST-S-3 specimen, featuring perforated support plates, were 4455.1 kN and 4410.7 kN, respectively. These values represent increases of approximately 9.4% and 8.3% compared to the peak load of 4071.3 kN observed for the CFST-M-2 specimen. This enhancement suggests that the inclusion of support plates, which divide the steel tube into three independent chambers, significantly improves the confinement effect of the steel tube on the concrete. Consequently, this modification enhances the load distribution and increases the overall load-bearing capacity of the structure.

**Figure 10:**
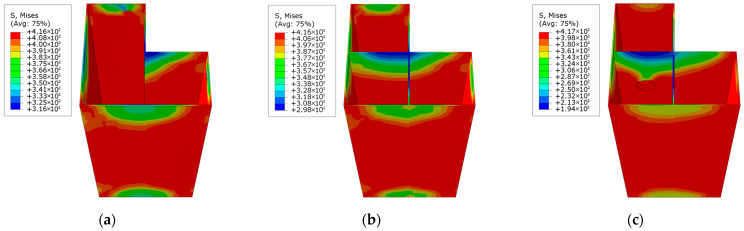
Stress distribution contour map of the steel tube at 85% of the peak load: (**a**) CFST-M-2; (**b**) CFST-S-2; (**c**) CFST-S-3.

The loading process of L-shaped CFST columns can be divided into three distinct stages: the elastic stage, the elastoplastic stage, and the failure stage. During the elastic stage, the displacement of all L-shaped CFST columns increases linearly with the applied load, as depicted in Figure 9. At the conclusion of this stage, the stress in the steel tube is uniformly distributed along the midsection of the column, reaching the yield stress of the steel, as shown in Figure 10. In contrast, the stress at the upper and lower ends of the column remains relatively low and has not yet reached the yield point. This observation indicates that, under compression, the out-of-plane buckling of the steel tube predominantly occurs in the midsection of the column, while the ends exhibit minimal deformation and do not experience buckling. The stress distribution in the CFST-S-2 specimen closely resembles that of the CFST-S-3 specimen. However, the CFST-S-2 specimen exhibits a slightly larger region at the column ends where the yield stress is reached. Notably, the presence of holes in the support plates does not significantly affect the stress distribution during the elastic stage.

**Figure 11:**
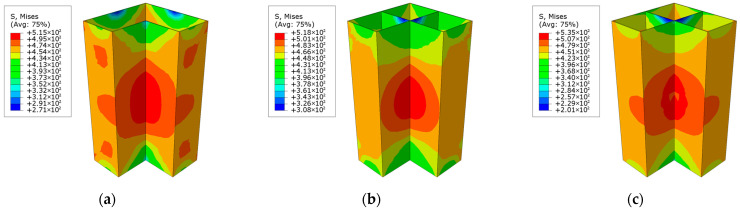
Stress distribution of steel tube at maximum displacement: (**a**) CFST-M-2; (**b**) CFST-S-2; (**c**) CFST-S-3.

As illustrated in Figure 11, at the maximum displacement, the high-stress region on the outer surface of the steel tube in the CFST-M-2 model (without support plates) is more extensive compared to the CFST-S-2 and CFST-S-3 models. In the CFST-M-2 model, the midsection of the concave corner is particularly prone to buckling. Additionally, minor stress concentration is observed on the two short sides of the L-shaped column, which may contribute to the local bulging of the steel tube. In contrast, the CFST-S-2 and CFST-S-3 models, which incorporate two support plates and perforated support plates, respectively, exhibit a more uniform stress distribution on the outer surface of the steel tube. The inclusion of perforations in the support plates reduces their stiffness, enhancing their ability to participate in energy dissipation. This modification effectively mitigates the likelihood of local buckling at the concave corners of the L-shaped CFST columns, thereby improving their structural performance.

### 4.2. Performance Analysis Under Unidirectional Eccentric Load

**Figure 12:**
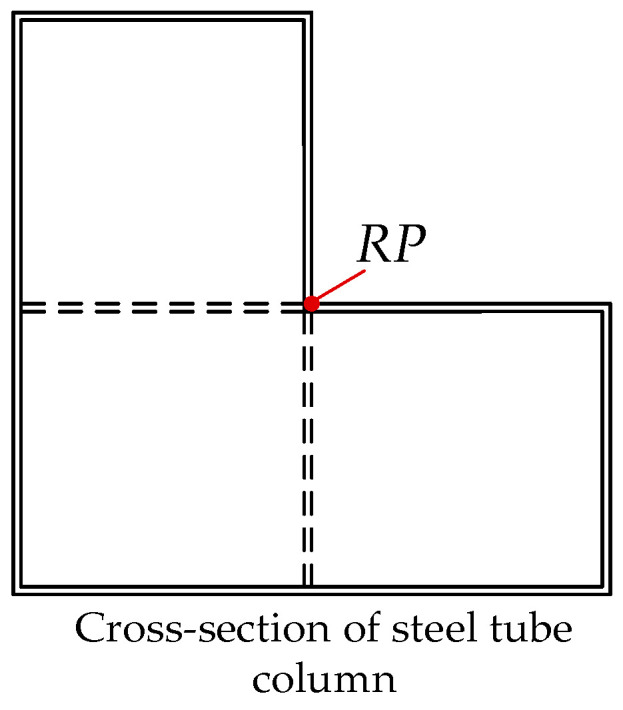
Location of unidirectional eccentric load.

The unique structural form of irregular L-shaped CFST columns necessitates an in-depth investigation into their compressive performance under eccentric loading. Finite element simulations were conducted on three models—CFST-M-2, CFST-S-2, and CFST-S-3—to analyze their stress behavior under such conditions. Given the distinctive geometry of the irregular column cross-section, the *RP* point (as shown in Figure 12) was selected as the loading application point for vertical loads.

**Figure 13:**
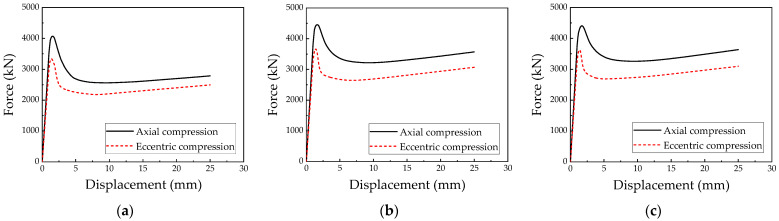
Comparison of load–displacement curves under eccentric and axial compression conditions: (**a**) CFST-M-2; (**b**) CFST-S-2; (**c**) CFST-S-3.

Figure 13 illustrates the load–displacement curves for the irregular L-shaped CFST columns under both axial and eccentric loading conditions. The simulation results reveal that the peak loads for the CFST-M-2, CFST-S-2, and CFST-S-3 models under unidirectional eccentric loading are 3339.3 kN, 3664.4 kN, and 3632.3 kN, respectively, corresponding to approximately 82.0%, 82.2%, and 82.3% of their ultimate axial compressive loads. These findings demonstrate that the inclusion of support plates significantly enhances the ultimate bearing capacity of irregular L-shaped CFST columns under eccentric loading. Furthermore, the presence of perforations in the support plates has a negligible effect on this capacity, suggesting that their influence can be reasonably disregarded in design considerations.

**Figure 14:**
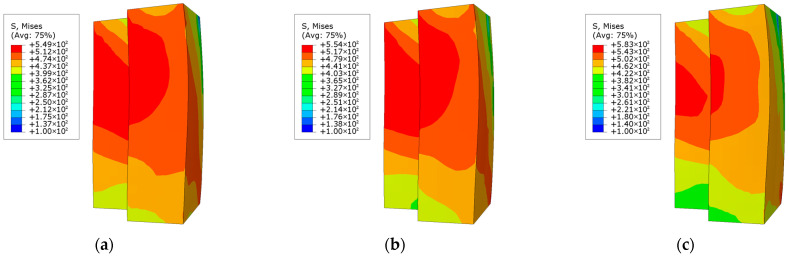
Stress distribution of steel tube at column end at maximum displacement under eccentric load: (**a**) CFST-M-2; (**b**) CFST-S-2; (**c**) CFST-S-3.

Figure 14 illustrates the stress distribution at the maximum displacement of the L-shaped steel tube at the column end. The results reveal that the upper section of the concave corner of the L-shaped steel tube exhibits relatively high stress, accompanied by significant bending deformation in this region. The results suggest that all three specimens are likely to experience steel tube buckling at this location. In the CFST-M-2 model, the high-stress region of the steel tube is more extensive compared to the CFST-S-2 and CFST-S-3 models, which include support plates. This indicates that the steel tube in the CFST-M-2 model is more prone to premature buckling failure. In contrast, the CFST-S-3 model, which incorporates perforated support plates, demonstrates reduced stiffness in the plates. This adjustment allows the support plates to absorb a greater portion of the load, thereby alleviating the stress on the outer side of the steel tube. Consequently, the distribution of high-stress areas in the steel tube is more localized, effectively suppressing the onset of buckling to some extent.

**Figure 15:**
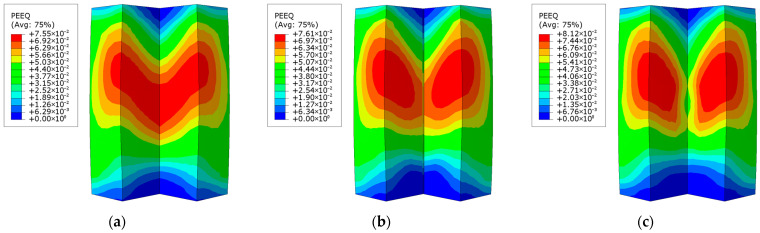
Distribution of equivalent plastic strain in the concrete column at the maximum displacement at the column base under eccentric loading: (**a**) CFST-M-2; (**b**) CFST-S-2; (**c**) CFST-S-3.

Figure 15 depicts the distribution of the equivalent plastic strain in the concrete sections of the three irregular L-shaped CFST columns. Among the models, CFST-M-2 exhibits the highest equivalent plastic strain, followed by CFST-S-2, while CFST-S-3 demonstrates the lowest strain values. This suggests that the concrete in the irregular column without support plates (CFST-M-2) undergoes more pronounced expansion. In regions with elevated equivalent plastic strain, the concrete in the CFST-M-2 model is more likely to exhibit V-shaped cracking near the upper section of the concave corner, ultimately leading to concrete failure. In the CFST-S-2 model, the addition of support plates effectively divides the concrete into separate chambers, forming three independent short concrete column segments.

As shown in Figure 15b, the V-shaped cracking in the upper section of the concave corner remains evident. However, the presence of the support plates provides restraint, resulting in narrower cracks at the midsection and wider openings at the end. In the CFST-S-3 model, while the concrete is similarly segmented by the support plates, the perforations allow the chambers to interconnect, maintaining the structural behavior of an L-shaped concrete column with embedded perforated support plates.

From the equivalent strain distribution in Figure 15c, regions of high plastic strain are observed in the upper concave corner of the concrete column. Unlike the CFST-M-2 and CFST-S-2 models, these high-strain areas do not form continuous regions, indicating that V-shaped cracking is unlikely to occur during actual testing. The reduced stiffness of the perforated support plates allows them to absorb more deformation, mitigating cracking at the middle corners of the L-shaped concrete column.

## 5. Parametric Analysis

### 5.1. Steel Strength

**Figure 16:**
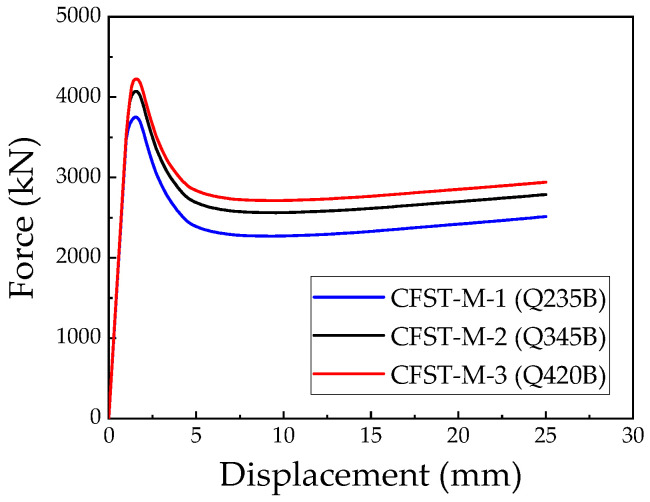
Load–displacement relationship in L-shaped CFST columns with different steel strengths.

The steel grades used in the models CFST-M-1, CFST-M-2, and CFST-M-3 are Q235B, Q345B, and Q420B, respectively, with all other simulation parameters kept constant, as detailed in Table 2. The corresponding load–displacement curves are presented in Figure 16. During the elastic stage, the initial stiffness of all specimens is nearly identical. However, the CFST-M-1 model, with the lowest steel strength, transitions to the elastoplastic stage earlier. All three models exhibit excellent compressive performance, characterized by a notable decline in load-bearing capacity after reaching the ultimate load. The CFST-M-1 model shows a more significant post-peak capacity reduction, with an average post-peak bearing capacity of approximately 58.3% of its ultimate load. In contrast, the CFST-M-2 and CFST-M-3 models maintain higher post-peak bearing capacities of around 65.2% and 67.3%, respectively. These results demonstrate the positive correlation between the steel strength and the compressive performance of irregular L-shaped CFST columns beyond their ultimate load.

Comparing the models, the ultimate bearing capacity of the CFST-M-2 model increased by approximately 8.5% relative to CFST-M-1, while the CFST-M-3 model achieved an increase of about 12.6%. Additionally, the bearing capacity enhancement factor (*δ*) in Table 2 reveals a consistent upward trend with increasing steel strength. This trend highlights the role of higher-strength steel in enhancing the confinement effect on the core concrete, thereby improving the compressive bearing capacity of the L-shaped CFST columns.

**Figure 17:**
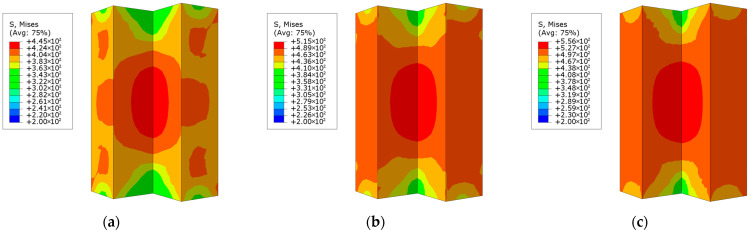
Stress distribution of steel tube at column end at maximum displacement under eccentric load: (**a**) CFST-M-1; (**b**) CFST-M-2; (**c**) CFST-M-3.

Figure 17 illustrates the stress distribution of the steel tube in the L-shaped CFST columns at the maximum displacement for different steel strengths. In the CFST-M-1 model, which utilizes the lowest-strength steel, the stress distribution is highly uneven, with significant stress concentration in the concave corner of the L-shaped column. This uneven distribution increases the likelihood of localized buckling in the steel tube, potentially leading to premature structural failure. Conversely, the CFST-M-2 and CFST-M-3 models, which employ higher-strength steels, exhibit more uniform stress distributions. Although the highest stress remains concentrated in the concave corner, the stresses in other regions are also elevated, resulting in a more balanced loading state. This uniform stress distribution facilitates the coordinated expansion of the steel tube and the encased concrete, enhancing the structural integrity of the columns. The use of higher-strength steel effectively mitigates localized buckling in the concave corner, improving the overall stability and performance of the L-shaped CFST columns under maximum displacement conditions.

In Table 2, *N_u_* is the nominal load-bearing capacity, and *N_u_* = *f_y_A_s_* + *f_ck_A_c_*, where *f_y_* is the yield strength of the steel and *A_s_* is the cross-sectional area of the steel tube; *f_ck_* is the characteristic compressive strength of concrete; *A_c_* is the cross-sectional area of the concrete column; and *δ* is the load-bearing capacity enhancement factor of the CFST column, where *δ* = *(N_u_* − *N_um_)/N_u_*, and *N_um_* is the simulated ultimate load capacity.

### 5.2. Steel Tube Thickness

**Figure 18:**
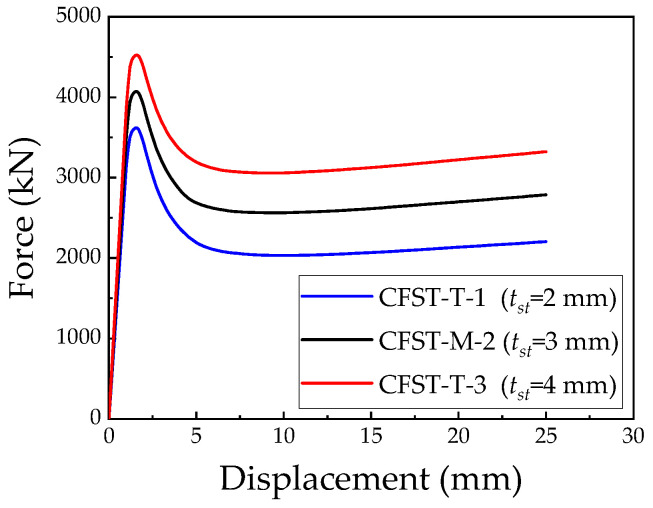
Load–displacement relationship for L-shaped CFST columns with different steel tube thicknesses.

Numerical simulations were carried out on L-shaped CFST columns with varying steel tube thicknesses of 2 mm, 3 mm, and 4 mm to assess their ultimate bearing capacity, as depicted in Figure 18. Table 3 summarizes the simulation parameters for the L-shaped CFST columns with different steel tube thicknesses. As shown in Figure 18, the load–displacement curves for all models exhibit similar trends before reaching the ultimate load. Notably, both the stiffness and bearing capacity improve significantly with an increasing steel tube thickness. The peak load achieved by the CFST-T-1 column (*t* = 2 mm) is approximately 3618.7 kN, while the CFST-M-2 column (*t* = 3 mm) reaches approximately 4071.3 kN, and the CFST-T-3 column (*t* = 4 mm) reaches approximately 4525.6 kN. In the post-peak plastic failure stage, the load–displacement curves stabilize at approximately 58.1%, 65.2%, and 68.8% of the ultimate load for the CFST-T-1, CFST-M-2, and CFST-T-3 models, respectively. These results highlight that increasing the steel tube thickness enhances both the ultimate bearing capacity and the post-peak residual capacity of the L-shaped CFST columns. In practical applications, employing slightly thicker steel tubes can effectively improve the compressive performance of CFST hybrid columns, contributing to greater structural stability and reliability.

The bearing capacity increase factor (*δ*) for the CFST-T-1, CFST-M-2, and CFST-T-3 models decreases progressively with an increasing steel tube thickness. This observation suggests that, while increasing the steel tube thickness enhances the ultimate bearing capacity of L-shaped CFST columns, the incremental improvement in the bearing capacity diminishes as the thickness continues to increase. This finding highlights a practical limitation: although thicker steel tubes improve the structural performance, the marginal benefits in terms of the bearing capacity become less significant with further increases in thickness. Consequently, in practical engineering applications, the decision to increase the steel tube thickness should be balanced against the associated fabrication costs and diminishing returns in performance enhancement.

**Figure 19:**
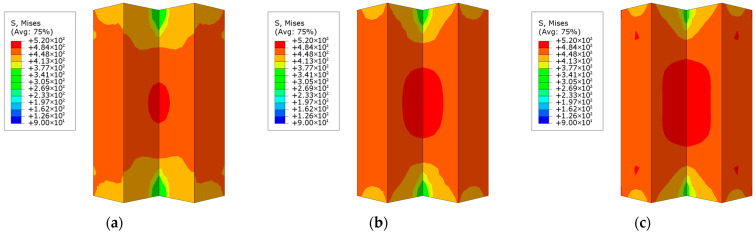
Stress distribution of steel tube at maximum displacement: (**a**) CFST-T-1; (**b**) CFST-M-2; (**c**) CFST-T-3.

The stress distribution of the steel tube at the maximum displacement, as illustrated in Figure 19, reveals that thinner steel tubes exhibit relatively concentrated stress, making the concave corners of the L-shaped CFST columns more susceptible to buckling. Conversely, as the steel tube thickness increases, the stress distribution becomes more uniform, effectively mitigating the premature buckling failure of the steel tube walls. This demonstrates that increasing the steel tube thickness is a viable strategy to suppress wall buckling, thereby enhancing the structural performance of the column. Overall, a greater steel tube thickness significantly improves the stiffness of the specimen, moderately increases the ultimate bearing capacity of the CFST column, and reduces localized buckling and other failure modes, demonstrating a pronounced structural reinforcement effect.

### 5.3. Number of Support Plates

**Figure 20:**
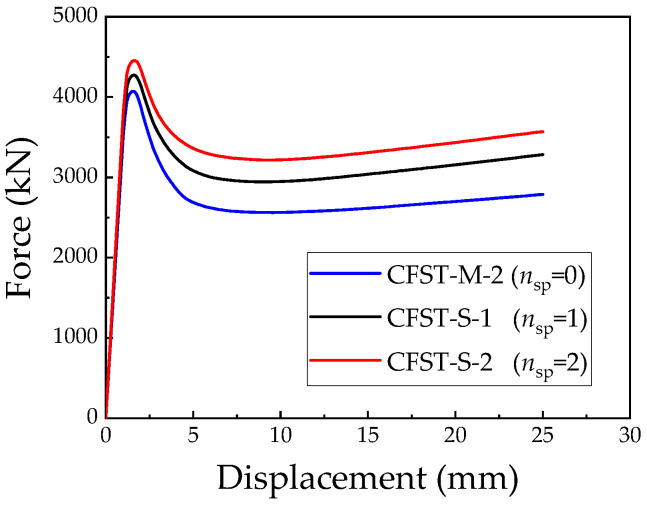
Load–displacement relationship for L-shaped CFST columns with different numbers of supporting plates.

The numerical simulation results and comparative analysis of L-shaped CFST columns with varying numbers of support plates (*n_sp_*) are presented in Table 4 and Figure 20. From the results, it is evident that the addition of support plates does not significantly influence the ascending or residual segments of the load–displacement curves for columns with one or two support plates. However, a marked effect is observed in the descending segment of the curves.

The CFST-S-1 model, incorporating one support plate, achieves an ultimate load-bearing capacity that is approximately 4.9% higher than that of the CFST-M-2 model, which lacks support plates. Similarly, the CFST-S-2 model, featuring two support plates, exhibits an ultimate load-bearing capacity that is about 9.4% greater than that of the CFST-M-2 model. Upon reaching the ultimate load-bearing capacity, the load–displacement curves transition into the descending segment, where the rate of load reduction decreases with an increasing number of support plates. Specifically, the curves for the CFST-M-2, CFST-S-1, and CFST-S-2 models stabilize at 65.2%, 73.0%, and 74.9% of their respective ultimate loads. This demonstrates that the inclusion of support plates effectively mitigates the rate of load reduction and enhances the compressive performance of L-shaped CFST columns after reaching their peak load.

A comparison of the bearing capacity enhancement factor (*δ*) in Table 4 further highlights the positive effect of support plates in improving *δ*, thereby enhancing the confinement provided by the steel tube to the concrete core.

**Figure 21:**
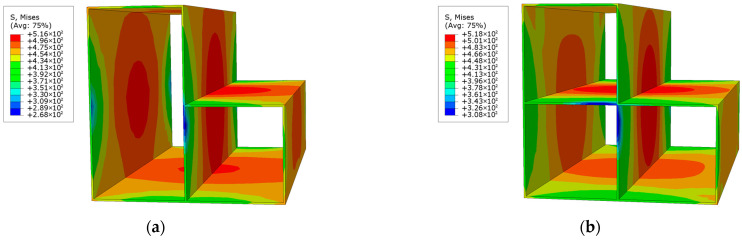
Stress distribution of steel tube at maximum displacement: (**a**) CFST-S-1; (**b**) CFST-S-2.

Figure 21 illustrates the stress distribution of the L-shaped CFST columns at the maximum displacement. The stress region exceeding 500 MPa is notably larger in the CFST-S-1 model with one support plate than in the CFST-S-2 model with two support plates. Stress concentration is observed at the centers of the cross-sections and the concave corners of both models, rendering these regions more susceptible to steel tube bulging. However, the second support plate in the CFST-S-2 model reduces the stress in the long-side steel tube wall compared to the CFST-S-1 model, effectively suppressing bulging in this area. The additional constraint provided by the second support plate significantly enhances the structural stability of the L-shaped CFST column.

### 5.4. Perforation of Support Plates

In irregular L-shaped CFST columns, the installation of support plates has been demonstrated to significantly enhance the column’s ultimate bearing capacity by improving the confinement effect of the steel tube on the concrete core. However, the inclusion of support plates divides the interior of the steel tube into multiple independent chambers, necessitating the separate pouring of concrete into each chamber. This process increases the complexity and workload of on-site construction, posing a practical challenge. To address this issue, this section investigates the impact of perforating the support plates on the compressive performance of L-shaped CFST columns. The proposed perforations interconnect the previously independent chambers within the steel tube, thereby eliminating the need for multiple concrete pours while retaining the confinement benefits provided by the support plates.

The load–displacement behavior of the CFST-S-3 model, which incorporates perforated support plates, is presented in Figure 9. The results reveal that the introduction of perforations has a minimal impact on the ultimate bearing capacity of L-shaped CFST columns with two support plates. The ultimate bearing capacity of the CFST-S-3 model is approximately 44.8 kN lower than that of the CFST-S-2 model with unperforated support plates, a difference that is considered negligible given the overall load scale. Moreover, when examining the post-peak load behavior, the CFST-S-3 model exhibits slightly superior performance compared to the CFST-S-2 model. Specifically, the compressive bearing capacity of the CFST-S-2 model decreases to approximately 74.9% of its peak load after reaching the ultimate load, whereas the CFST-S-3 model remains at around 76.3% of its peak load. This finding suggests that perforated support plates not only maintain the column’s ultimate bearing capacity but also enhance its compressive strength and stability in the post-peak load stage.

**Figure 22:**
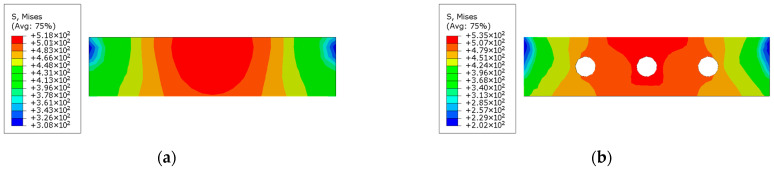
Stress distribution of the supporting plate: (**a**) CFST-S-2; (**b**) CFST-S-3.

Further insights can be derived from the stress contour plots of the support plates at maximum displacement, shown in Figure 22. These plots indicate that the yield stress area of the perforated support plate is significantly larger than that of the unperforated plate. In the perforated support plate, high-stress regions are primarily concentrated around the circular holes, with the maximum stress reaching critical levels in these areas. In contrast, the unperforated support plate exhibits a maximum stress value of approximately 480 MPa, but this stress is distributed over a relatively smaller area. The concentrated stress distribution around the perforations suggests that the perforated support plates effectively contribute to energy dissipation under loading conditions.

Overall, the findings indicate that perforating the support plates is a practical and effective modification to L-shaped CFST columns. The perforations allow for interconnected chambers within the steel tube, thereby simplifying construction processes and reducing the on-site workload. At the same time, the structural integrity and performance of the columns are preserved, with potential enhancements in the post-peak compressive strength and energy dissipation capabilities. The larger yield stress area and concentrated stress regions around the perforations suggest improved structural resilience, making perforated support plates a favorable design feature. Consequently, it is recommended that perforations be incorporated into support plates in practical construction applications to enhance both the construction efficiency and the structural performance of L-shaped CFST columns.

### 5.5. Height of CFST Column

**Figure 23:**
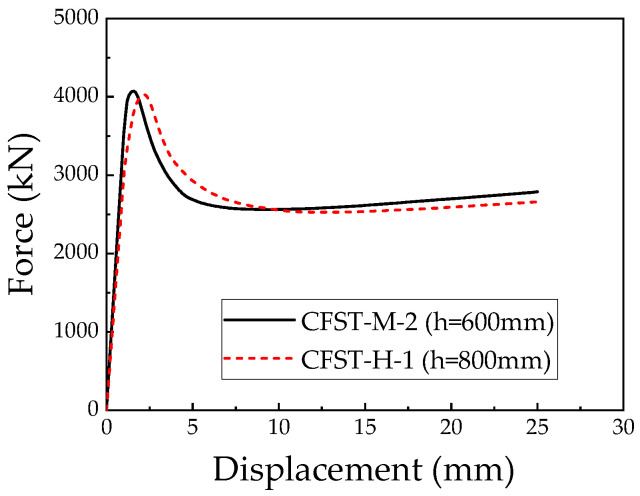
Comparison of load–displacement relationships for L-shaped CFST columns with different column heights.

Taking the CFST-M-2 specimen as the baseline model, the column height was adjusted to 800 mm to establish the CFST-H-1 model. The finite element analysis results, including the ultimate bearing capacity and the load–displacement relationship, are presented in Figure 23. The ultimate bearing capacity of the CFST-H-1 model is 4030.6 kN, which is 1.1% lower than that of the CFST-M-2 model, which has a column height of 600 mm. This indicates that reducing the column height has a marginally positive effect in terms of enhancing the ultimate bearing capacity, although the influence is relatively small.

**Figure 24:**
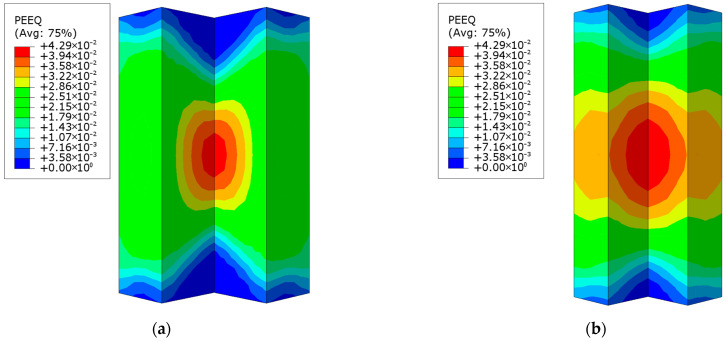
Equivalent plastic strain (PEEQ) distribution contour for L-shaped concrete columns: (**a**) CFST-M-2; (**b**) CFST-H-1.

As illustrated in Figure 24, the distribution of the equivalent plastic strain reveals that both the 600 mm and 800 mm column heights lead to relatively high equivalent plastic strain in the midsection of the concave corner of the L-shaped concrete column. For the 600 mm column, the region with high strain is more concentrated, indicating localized deformation. In contrast, for the 800 mm column, the high-strain region extends beyond the concave corner, affecting a larger area that includes the short-side surface of the concrete. This increase in the affected area suggests that a taller column leads to a more distributed and extensive strain development within the concrete. As the column height increases, the potential for concrete cracking also rises due to the greater strain accumulation. Therefore, in practical engineering design and construction, it is crucial to carefully control the column height to manage strain distribution and minimize the risk of cracking.

### 5.6. Partial Damage Analysis of Concrete

**Figure 25:**
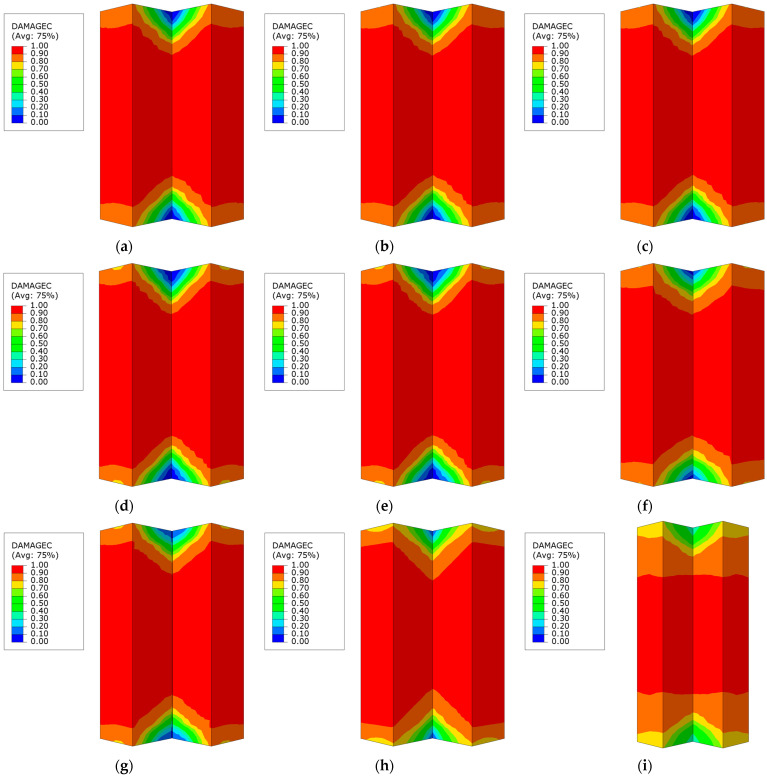
Concrete compression damage distribution: (**a**) CFST-M-1; (**b**) CFST-M-2; (**c**) CFST-M-3; (**d**) CFST-T-1; (**e**) CFST-T-3; (**f**) CFST-S-1; (**g**) CFST-S-2; (**h**) CFST-S-3; (**i**) CFST-H-1.

Figure 25 illustrates the compressive damage distribution in the concrete portion of the L-shaped CFST columns at the final failure stage, based on the finite element analysis. As shown, apart from the CFST-H-1 specimen with a column height of 800 mm, the overall compressive damage distribution in the specimens is relatively consistent: less damage is observed at both ends of the columns, severe damage occurs near the central core region, and the concave corner areas at the ends exhibit the least damage. This indicates that altering the steel strength or steel tube thickness or adding internal stiffener plates has a limited influence on the overall failure pattern of the concrete core.

A comparison of Figure 25b,i reveals that the compressive damage in the concrete is primarily concentrated in the mid-height region of the columns, and, as the column height increases, the extent of the most severely damaged zone decreases. Specifically, when the L-shaped CFST column reaches its ultimate failure stage, if the column height is relatively small, a large portion of the in-tube concrete undergoes compressive failure, potentially causing the crushing of the concrete surface. Conversely, if the column height is relatively large, the zone of compressive failure within the concrete core becomes smaller and is confined to the middle segment, which can lead to cracking in this region.

## 6. Conclusions

This study focused on the axial compressive performance of L-shaped CFST columns and examined the effects of installing support plates. The key research parameters included the steel strength, the pipe thickness, the number of support plates, and the presence of holes in the support plates. Nine finite element models were developed for parametric analysis, and the findings reveal the following.

1. The axial compression behavior of the L-shaped CFST column can be divided into three distinct phases: the elastic phase, the elastoplastic phase, and the failure phase. At the conclusion of the elastic phase, the steel pipe, except for the regions at the top and bottom ends, reaches its yield stress, resulting in a relatively uniform stress distribution within the steel pipe.
2. The ultimate bearing capacity of L-shaped CFST columns under eccentric loading is approximately 82% of that observed under axial compression. Eccentric loading induces a shift in the bulging position at the corner of the L-shaped steel tube, moving it upward. The installation of support plates enhances the ultimate bearing capacity of L-shaped CFST columns under eccentric loading. In the model without support plates (CFST-M-2) and the model with two support plates (CFST-S-2), V-shaped cracks develop at the upper part of the concave corner of the concrete column. However, in the model with perforated support plates (CFST-S-3), the equivalent plastic strain around the corner is effectively reduced, which helps to suppress crack propagation in the corner region.
3. As the steel strength grade increases, the initial stiffness and deformation capacity of the L-shaped CFST column remain largely unchanged. However, when the steel strength is upgraded from Q235B to Q345B and Q420B, the model’s ultimate bearing capacity increases by 8.5% and 12.6%, respectively. The enhancement in steel strength effectively improves the load-bearing capacity factor of the L-shaped CFST column, leading to the greater confinement of the concrete by the steel, thereby improving the overall load-bearing performance.
4. Compared with the CFST-T-1 model, which had a steel pipe thickness of 2 mm, the ultimate bearing capacity of the column models with steel pipe thicknesses of 3 mm and 4 mm increased by 12.5% and 25.1%, respectively. After entering the plastic failure stage, the load–displacement curves for the CFST-T-1, CFST-M-2, and CFST-T-3 models dropped to 58.1%, 65.2%, and 68.8% of their respective ultimate loads before stabilizing. Increasing the steel pipe thickness enhances the compressive capacity of the L-shaped CFST column after it reaches the ultimate bearing capacity.
5. The installation of support plates can effectively enhance the ultimate bearing capacity of the L-shaped CFST column. As the number of support plates increases, the rate of load decreases in the descending section of the load–displacement curve slows down, which can effectively reduce the load drop and improve the compressive capacity of the L-shaped CFST column after reaching its ultimate bearing capacity.
6. Perforating the support plates of the L-shaped CFST column, which connects the previously separated chambers, enhances the efficiency of concrete pouring. The simulation results indicate that the presence of holes has a minimal impact on both the ultimate bearing capacity and the compressive strength of the column after reaching its ultimate load. Under eccentric loading, the reduced stiffness of the perforated support plates allows them to bear a greater load. In areas where specific compressive bearing capacity requirements are not critical, perforating the support plates is recommended to improve the construction efficiency.
7. Reducing the column height of L-shaped CFST columns can improve their ultimate bearing capacity, although the effect is relatively modest. Conversely, increasing the column height raises the risk of cracking in the midsection of the concave corner. Therefore, in practical engineering applications, it is crucial to carefully control the column height to minimize the risk of concrete cracking and ensure structural integrity.

Our future work will focus on conducting numerical studies to investigate the effects of the hole type, hole quantity, and plate thickness in the internal support plates of the steel tube on the axial compressive performance of short columns. In addition, experimental analyses will be conducted to further explore the load-bearing capacity of L-shaped CFST columns, the buckling behavior of the steel tube, and the compressive failure mechanisms of the in-tube concrete. Moreover, the seismic performance of beam–column joints suitable for this type of CFST column will also be examined.

## Figures and Tables

**Table 1:** Summary of parameters for irregular L-shaped CFST columns.

Specimen	Steel Grade	Concrete	*t_st_* (mm)	*n_sp_* ^1^	Hole Configuration of SP	*H* ^2^ (mm)
CFST-M-1	Q235B	C40	3	0	/	600
CFST-M-2	Q345B		3	0	/	600
CFST-M-3	Q420B		3	0	/	600
CFST-T-1	Q345B		2	0	/	600
CFST-T-3	Q345B		4	0	/	600
CFST-S-1	Q345B		3	1	/	600
CFST-S-2	Q345B		3	2	/	600
CFST-S-3	Q345B		3	2	√	600
CFST-H-1	Q345B		3	0	/	800

^1^ Number of support plates (SP); ^2^ Column height.

**Table 2:** Parameters for L-shaped CFST columns with different steel strengths.

Specimen	Steel Grade	*N_u_* (kN)	*N_um_* (kN)	*δ*
CFST-M-1	Q235B	2966.9	3751.9	0.265
CFST-M-2	Q345B	2966.9	4071.3	0.372
CFST-M-3	Q420B	2966.9	4226.9	0.425

**Table 3:** Parameters for L-shaped CFST columns with different steel tube thicknesses.

Specimen	*t_st_* (mm)	*N_u_* (kN)	*N_um_* (kN)	*δ*
CFST-T-1	2	2583.6	3618.7	0.401
CFST-M-2	3	2966.9	4071.3	0.372
CFST-T-3	4	3347.7	4525.6	0.352

**Table 4:** Parameters for L-shaped CFST columns with different numbers of supporting plates.

Specimen	*n_sp_*	*N_u_* (kN)	*N_um_* (kN)	*δ*
CFST-M-2	0	2966.9	4071.3	0.372
CFST-S-1	1	3104.4	4274.5	0.377
CFST-S-2	2	3241.9	4455.1	0.374

## Data Availability

The original contributions developed in this study are included in this article; further inquiries can be directed to the corresponding authors.
